# Supplementary material for: Vaccination of Elms against Dutch Elm Disease—Are the Associated Epiphytes and Endophytes Affected?
Source: J Fungi (Basel). 2023 Feb 24;9(3):297. doi: 10.3390/jof9030297 (PMC10057572; doi:10.3390/jof9030297)
Supplement: Supplementary file 1 [file jof-09-00297-s001.zip › Figure S1_Vaccination of elms.pdf]

Supplementary figure

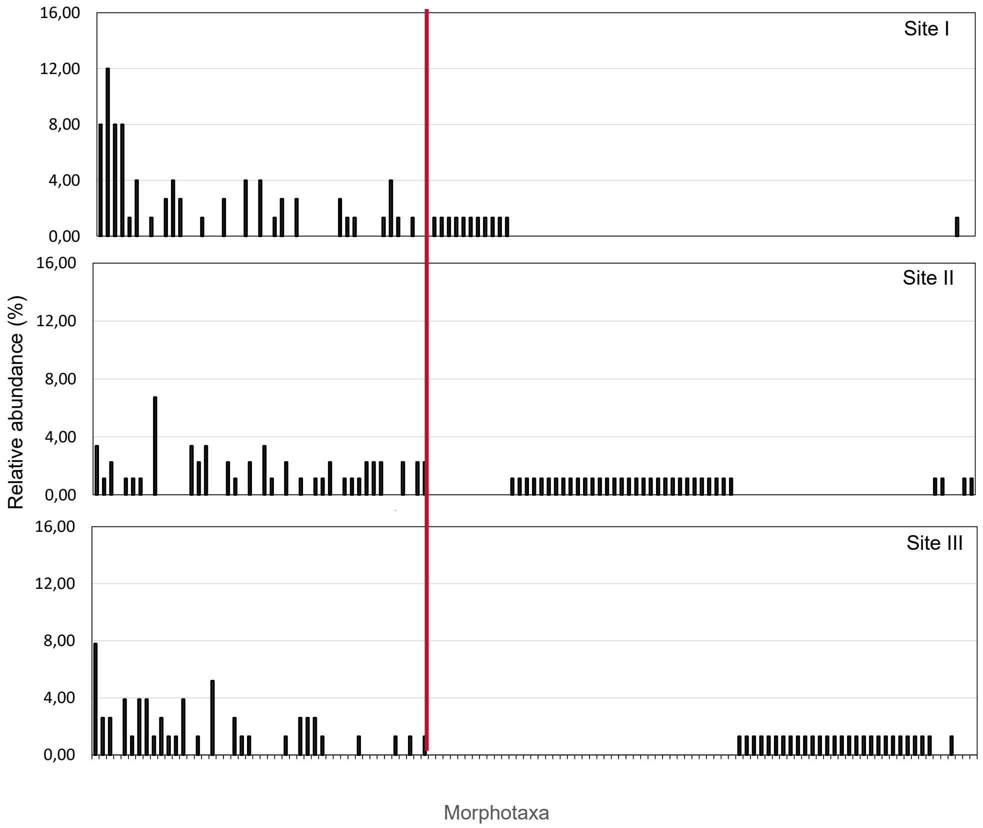

Figure S1. Bar chart showing the relative abundance (%) values for 21 individual morphotaxa detected in bark and xylem samples from elms growing at the three sites where the vaccinations using Dutch Trig ® were ongoing at the time point of sampling (Site I), where vaccinations were ceased (Site II) and where trees had not been vaccinated (Site III). On the left side of the vertical red line: morphotaxa nr 1 to 43, 119, 120 and 121 (in that order); on the right side of the vertical red line singleton morphotaxa 55 to 118, in ascending order.
