# Supplementary material for: Vaccination of Elms against Dutch Elm Disease—Are the Associated Epiphytes and Endophytes Affected?
Source: J Fungi (Basel). 2023 Feb 24;9(3):297. doi: 10.3390/jof9030297 (PMC10057572; doi:10.3390/jof9030297)
Supplement: Supplementary file 1 [file jof-09-00297-s001.zip › Table S1.pdf]

Supplementary table

Table S1. Percentage of epiphyte cover in unvaccinated and vaccinated elms (Study 1, Stockholm)

Tree ID	DBH	Treatment	N	E	S	W	Mean
22_11H_111	38,5	Unvaccinated	6,00%	9,0%	5,0%	1,0%	5,3%
22_11H_151	18,5	Unvaccinated	2,00%	0,0%	0,0%	0,0%	0,5%
22_11H_174	46,2	Unvaccinated	3,00%	0,0%	0,0%	0,0%	0,8%
22_11H_178	36,8	Unvaccinated	88,00%	66,0%	58,0%	24,0%	59,0%
22_11H_231	24,4	Unvaccinated	16,00%	9,0%	0,0%	4,0%	7,3%
22_11H_240	36,7	Unvaccinated	44,00%	62,0%	12,0%	24,0%	35,5%
22_11H_298	33,6	Unvaccinated	7,00%	2,0%	1,0%	2,0%	3,0%
22_11H_307	42,7	Unvaccinated	23,00%	10,0%	8,0%	3,0%	11,0%
22_19A5_1159	24,5	Unvaccinated	73,00%	47,0%	86,0%	32,0%	59,5%
22_19C_228	30,8	Unvaccinated	25,00%	11,0%	8,0%	9,0%	13,3%
22_19C_451	26,6	Unvaccinated	3,00%	0,0%	1,0%	5,0%	2,3%
22_19C_565	18,5	Unvaccinated	33,00%	28,0%	47,0%	77,0%	46,3%
22_19C_567	24,5	Unvaccinated	13,00%	5,0%	17,0%	78,0%	28,3%
22_19C_593	23,9	Unvaccinated	65,00%	21,0%	18,0%	42,0%	36,5%
22_20A_342	37,1	Unvaccinated	9,00%	15,0%	7,0%	3,0%	8,5%
22_20C_20	18,0	Unvaccinated	95,00%	88,0%	93,0%	77,0%	88,3%
22_20C_383	28,4	Unvaccinated	65,00%	22,0%	3,0%	16,0%	26,5%
22_11A_123	27,7	Vaccinated	37,00%	33,0%	0,0%	22,0%	23,0%
22_11A_59	23,0	Vaccinated	0,01%	12,0%	8,0%	18,0%	9,5%
22_11B_124	33,3	Vaccinated	9,00%	1,0%	1,0%	3,0%	3,5%
22_11B_129	39,8	Vaccinated	28,00%	3,0%	0,0%	14,0%	11,3%
22_11B_191	38,3	Vaccinated	12,00%	5,0%	4,0%	9,0%	7,5%
22_11B_203	29,8	Vaccinated	63,00%	56,0%	26,0%	18,0%	40,8%
22_11B_210	22,5	Vaccinated	2,00%	60,0%	74,0%	78,0%	53,5%
22_11B_248	40,7	Vaccinated	6,00%	0,0%	0,0%	0,0%	1,5%
22_11B_302	39,0	Vaccinated	29,00%	4,0%	0,0%	6,0%	9,8%
22_11C_307	29,6	Vaccinated	30,00%	6,0%	2,0%	6,0%	11,0%
22_11C_331	25,2	Vaccinated	7,00%	2,0%	1,0%	7,0%	4,3%
22_11C_34	20,9	Vaccinated	56,00%	24,0%	21,0%	8,0%	27,3%
22_11F_204	27,0	Vaccinated	5,00%	21,0%	14,0%	11,0%	12,8%
22_11F_217	36,6	Vaccinated	4,00%	3,0%	4,0%	2,0%	3,3%
22_11G_152	34,8	Vaccinated	18,00%	13,0%	21,0%	12,0%	16,0%
22_11G_284	34,4	Vaccinated	7,00%	6,0%	3,0%	2,0%	4,5%
22_11G_290	32,3	Vaccinated	2,00%	1,0%	16,0%	20,0%	9,8%
